# Diabetic retinopathy in rural communities: a review of barriers to access of care and potential solutions

**DOI:** 10.1080/07853890.2025.2522323

**Published:** 2025-07-04

**Authors:** James Burmeister, Madeline N. Pham, Forrest Bohler, Caleb North

**Affiliations:** a Department of Foundational Medical Studies, Oakland University William Beaumont School of Medicine, Rochester, MI, USA; b Mercer University School of Medicine, Savannah, GA, USA; c Montana State University, College of Letters and Science, Bozeman, MT, USA

**Keywords:** Diabetic retinopathy, rural healthcare, rural communities, Barriers in access to care, telehealth

## Abstract

**Introduction:**

Diabetic retinopathy (DR) is a leading cause of vision loss. With an estimated 38.4 million Americans diagnosed with DM, the disease exerts a significant burden on healthcare systems, especially in rural areas where access to care is limited. DR prevalence is notably higher in rural communities due to barriers such as geographical isolation, lower socioeconomic status, and provider shortages.

**Objective:**

This narrative review explores the current state of DR management in rural areas, highlighting the increased incidence of the condition in these regions and the unique challenges faced by rural patients.

**Barriers to Care:**

Key barriers to care include distance and travel, financial constraints, and a lack of ophthalmology and optometry specialists.

**Solutions:**

The review also discusses potential solutions to improve DR outcomes, including teleophthalmology, artificial intelligence (AI) screening, and expanding rural healthcare workforce programs. These interventions aim to improve early detection and access to treatment, ultimately reducing the disparity in DR care between rural and urban populations. Comprehensive efforts from policymakers, healthcare systems, and educational institutions will be crucial in addressing the gaps in rural DR care and improving patient outcomes.

## Introduction

Diabetes mellitus (DM) is a dysregulation in blood glucose levels resulting in chronic hyperglycaemia. Type 1 diabetes is an autoimmune disease resulting in the destruction of pancreatic beta cells leading to a lack of endogenous insulin production [1]. Although considered to be a more severe form of DM, Type 1 diabetes is only prevalent among 1.3 million Americans [2]. Type 2 diabetes, also known as insulin-resistant diabetes, is due to a dysregulated response to insulin from years of poorly managed chronic hyperglycaemia and represents the vast majority of diagnosed DM [2]. The common thread between these types of DM is chronically elevated blood glucose levels, leading to hyperglycaemia and its damaging downstream affects. Due to its high prevalence, DM and its associated health conditions exert a heavy financial toll on the country’s healthcare system. On average, those with DM utilize approximately $20,000 worth of healthcare costs a year, 60% of which can be directly attributable to DM costing the nation $327.2 billion a year in DM-related expenditures [3,4].

Of the 11.6% of the country’s population with DM, 8.7 million have yet to be diagnosed [3]. Delays in diagnosis can lead to debilitating complications of DM such as diabetic neuropathy diabetic nephropathy, diabetic retinopathy (DR) [5,6]. DR is now the leading cause of vision loss in the developed world among working age people [7]. Of those with DM, 26.43% suffer from DR in varying stages [8]. Virtually all patients diagnosed with Type 1 DM have developed some degree of DR within two decades of their diagnosis [9]. Within this same time frame, 60% of those with Type 2 DM will have DR [10].

One of the most important prognostic factors in the development and progression of DR is early identification and intervention of the disease [11,12]. This intervention often begins at the time of DM diagnosis as annual dilated eye exams, which are recommended within the first five years of Type 1 DM diagnosis and beginning immediately for those with Type 2 DM [13,14]. The presence of DR in 21% of patients at the time of their initial diagnosis of Type 2 DM, underscores the need for routine eye exams [9].

Although DM is a nation-wide epidemic, rural communities in the United States suffer from a 17% higher prevalence of diabetes and a disproportionately elevated diabetes-related mortality rate compared to urban areas [15–17]. Unsurprisingly, complications from DM, such as DR, are also more prevalent within these communities. For example, a national study found that patients in rural areas were significantly less likely to receive annual dilated eye exams than their urban counterparts [17]. Given that DR represents a serious health concern in an already marginalized population, it is crucial to identify the current barriers to care for this patient population [17]. In Native American populations, where diabetes is particularly prevalent, DR screening rates are even lower, compounding existing healthcare disparities and highlighting how geographic and systemic inequities exacerbate DR outcomes [18,19]. Given that DR represents a serious health concern in an already marginalized population, it is crucial to identify and address the unique barriers such as long travel distances to specialists, limited insurance coverage, and workforce shortages that rural patients face. This review outlines the current state of DR eye care in rural communities and identifies potential solutions to improve patient outcome for those with DR.

## Diabetic retinopathy treatment

Management of DR is multimodal with interventions sculpted to match the severity of the disease including routine intraocular injections, laser photocoagulation, and surgical interventions. The aims of treatment are principally to diminish or prevent the disease from worsening, retain sight, and in certain circumstances regain vision that has been lost.

## Anti-VEGF intraocular injections

The recommended cornerstone treatment for DR in developed countries, especially for Diabetic Macular Edema (DME) and Proliferative Diabetic Retinopathy (PDR), are intravitreal injections of anti-vascular endothelial growth factor (anti-VEGF) [20]. Anti-VEGF therapies include agents targeted at blocking the growth and development of abnormal blood vessels in the retina, while inhibiting vascular permeability that can result in fluid accumulation in the macula [21].

The frequency of anti-VEGF injections may vary based on the specific medication administered and individual patient response to treatment [21]. Initially, patients may require monthly injections over the course of several months [22]. Depending on patient compliance, the frequency of injections can typically be decreased to every 6–8 weeks following this loading phase or at even longer intervals if clinically appropriate [23]. However, many patients require ongoing treatments for extended periods of times, sometimes for years to sustain the beneficial outcomes [24].

The effort and time required by patients for this treatment is also substantial. Regular visits to specialized eye clinics, often located far from rural communities, are necessary. Many of these visits include an injection into one or both eyes as well as a pre-injection evaluation and post-injection observation. For patients in rural communities, this means significant travel time, potential work rescheduling/loss of work hours, and the need for transportation assistance which presents significant obstacles to encouraging frequent care in rural patients.

## Other treatment modalities

Although anti-VEGF injections are used as the primary form of treatment for DR, other therapies may be indicated depending on the manifestations within the individual patient. Laser photocoagulation is an effective treatment for PDR to lower retinal oxygen demand and help prevent severe vision loss [25]. If these treatments and therapies fail, people with severe cases of DR that present with significant bleeding into their vitreous or severe scar tissue formation may need a surgical intervention called vitrectomy [26]. A vitrectomy involves removing the vitreous gel and any scar tissue, potentially combined with laser treatment [27,28]. For instances of DME that are resistant to anti-VEGF therapy, corticosteroid injections into the eye can reduce inflammation and swelling in the retina. In addition, the management of concurrent diabetes has enormous importance in the overall care plan, including controlling and monitoring blood glucose, blood pressure, and lipids. This systematic screening is important in order to reduce the rate of progression of DR [29].

## Rural DR disease burden

Many studies have highlighted the unique challenges faced by rural communities in managing DR. One study based in rural India found that among patients with DM, the prevalence of DR was 10.3%, providing insight into the increased prevalence of DR in rural communities as a whole [30]. When researchers surveyed patients living with diabetes in rural China, they found that DR screening is underperformed due to unaffordability of medical costs, lack of medical facilities, and limited access to screening programs [31]. In addition, it was found that the prevalence of DR among adults with type 2 diabetes is higher in rural areas (29.1%) than in urban areas (18.1%) of China, further highlighting the urban vs. rural gap in DR care [31]. Another study conducted in the U.S. demonstrated that rural areas had significantly lower rates of annual eye examination for patients with DM when compared to urban regions and therefore may be at great risk of underdiagnosis or delayed treatment for DR [17 In rural Native American communities, where there is already an increased prevalence of DM, those with the disease were less likely to receive routine DR screening and treatment compared to their urban counterparts [18,19]. In totality, these studies demonstrate the marked rural-urban divide in DR care access and outcomes, underscoring the need for interventions to improve both screening efforts and treatment accessibility.

## Barriers to care in rural communities: a social ecological perspective

### Individual-level barriers

Rural areas typically have lower socioeconomic status (SES), which is associated with unhealthy eating habits and an increased risk of developing diabetes [32]. A study by the Rural Health Research Centre found that rural Americans are more likely to be obese and make poor dietary choices compared to their urban counterparts, partly due to limited access to healthy food options and lower income levels [33,34].

The intersection of low SES, inadequate insurance, and high treatment costs in a rural setting creates significant obstacles for patients with DR. Frequent travel every 3 to 6 months for treatments becomes impractical, and without adequate insurance coverage, accessing consistent care becomes an even greater challenge.

### Community-level barriers

There is a significant disparity in the distribution of eye care professionals, with a strong concentration in urban areas and minimal presence in rural communities. Although precise numbers are difficult to determine, rural areas have consistently faced a shortage of ophthalmologists and optometrists. One study found that among 187 emergency departments surveyed, only 48.6% of rural facilities had ophthalmology coverage, compared to 74.7% of non-rural facilities [35]. Additionally, rural facilities reported an average distance of 23.72 miles to a referral location, compared to just 4.41 miles for non-rural facilities [35]. Rural emergency departments often struggle with limited resources compared to their non-rural counterparts, resulting in more significant gaps in specialized care, particularly in ophthalmology.

Optometrists, who are often the initial point of contact for patients with DR, also face similar disparities. Although their representation is somewhat better than ophthalmologists, rural areas still have significantly reduced access to these providers. One report highlights that rural counties continue to suffer from a lack of availability of eye care professionals such as ophthalmologists and optometrists [36]. The scarcity of providers, combined with challenges in accessing timely care, increases the likelihood of delayed diagnoses and poorer outcomes for rural patients with DR.

Moreover, in rural areas, distance and travel present significant obstacles for DR patients, who must journey long distances to access specialized services. This challenge is not unique to DR; the literature consistently demonstrates similar barriers across many areas of healthcare. For instance, rural breast cancer patients are significantly less likely to undergo breast reconstruction surgery following mastectomy due to the limited availability of specialists in proximity [37]. Similarly, individuals with chronic diseases residing in remote areas have restricted access to regular specialist reviews, leading to poorer health outcomes. Rural residents experiencing mental health issues also face difficulties in obtaining psychiatric services due to the scarcity of professionals providing such care outside urban centres [38]. These examples illustrate a recurring pattern: the greater the distance to specialized care, the less likely patients are to receive essential treatments across various medical fields. For conditions like DR, where treatments must be administered regularly, on what can be a monthly basis, these barriers make it particularly challenging for rural patients to maintain consistent eye care [39].

### Systemic and policy-level barriers

Moreover, lower SES in rural areas correlates with lower rates of insurance coverage. According to the U.S. Census Bureau, rural areas have consistently higher uninsured rates than urban regions [40]. This lack of coverage poses a significant financial barrier to treatments like anti-VEGF therapies, which are crucial for DR. Without insurance, the cost of injections can be prohibitively expensive, reaching thousands of dollars per dose. For instance, a single injection of Eylea (aflibercept) can cost approximately $1,850 [41]. The combination of lower SES, limited insurance coverage, and the need for costly, repeated treatments makes managing DR particularly challenging for rural residents.

The lack of regular eye exams and limited awareness of DR screening in rural areas often leads to delayed diagnoses. A study conducted in Wisconsin found notable disparities in diabetic eye screening between rural and urban settings. Even after accounting for health system differences, patients at urban clinics were significantly more likely to receive screening compared to those at rural clinics [42].

This delay in early detection often means that rural patients are diagnosed at more advanced stages of the disease, reducing treatment options and resulting in poorer outcomes. The American Diabetes Association recommends that patients with Type 1 diabetes receive a comprehensive eye exam within five years of diagnosis, while patients with Type 2 diabetes should have an eye exam at the time of diagnosis [43]. Unfortunately, these guidelines are rarely adhered to in rural areas, contributing to less effective disease management and higher rates of preventable vision loss.

Although the literature on private equity in rural ophthalmology care is somewhat limited, the broader trend of private equity acquisitions in healthcare may have significant implications for rural eye care. Investor groups have increasingly targeted ophthalmology practices, acquiring them with an emphasis on consolidating operations [44].

This trend could have mixed effects on rural eye care. On one hand, private equity involvement could potentially lead to increased efficiency and more resources being directed to underserved rural areas [45]. On the other hand, there are concerns that the profit-driven nature of private equity may lead to cuts in services in less profitable rural locations, further exacerbating existing healthcare disparities [46].

## Potential solutions

### Telehealth initiatives

Telehealth, particularly teleophthalmology, has emerged as a promising solution to bridge the gap in rural eye care for DR. These services enable remote consultations and screenings, significantly reducing the need for frequent travel—a major obstacle for rural patients [47]. Research has shown that teleophthalmology can effectively screen for DR and increase screening rates in rural communities [48].

Building a robust telehealth infrastructure could alleviate many challenges associated with rural eye care. First, mobile screening units can enhance access to care for remote populations [49]. Second, virtual consultative services can help minimize unnecessary travel for follow-ups and treatment with retina specialists. Lastly, home monitoring devices capable of detecting visual changes and alerting healthcare providers can also contribute to better DR management [50]. Implementing telehealth programs in rural areas could greatly improve access to early diagnosis and treatment, ultimately enhancing patient outcomes. However, scalability depends heavily on broadband access, workforce training, and initial capital investment. In many U.S. rural counties, broadband coverage remains below national standards, limiting telehealth’s reach. Moreover, successful adoption requires sustained funding and alignment with reimbursement policies. For example, the Centres for Medicare & Medicaid Services (CMS) have expanded telehealth billing codes since the COVID-19 pandemic, which may provide the necessary infrastructure for broader implementation but only if these changes become permanent.

### Artificial intelligence (AI) screening

There are numerous opportunities to develop and validate AI-based screening tools for the early detection of DR in rural populations. These tools could be deployed in community settings allowing for initial screenings without the need for specialist involvement. One crucial element is the deployment of AI-powered retinal scanning devices in easily accessible locations such as pharmacies, community health centres, and other rural healthcare facilities. This could support the development of integrated referral systems capable of not only detecting DR but also automatically generating timely referrals for follow-up care when needed [51,52]. Specialists could remotely validate diagnoses and formulate treatment plans. Additionally, incorporating patient education modules into the AI screening pipeline could raise awareness of DR and emphasize the importance of follow-up care [53]. By introducing AI-driven screening tools in rural areas, early detection rates for DR could significantly increase, ultimately improving clinical outcomes for patients in these underserved communities.

### Rural incentive programmes

Expanding loan repayment programs to include ophthalmology could incentivize more providers to practice in rural areas. Currently, programs like the National Health Service Corps (NHSC) Loan Repayment Program primarily target primary care providers [54]. Creating similar programs specifically for ophthalmologists and retina specialists could help address the longstanding shortage of these specialists in rural communities. Additionally, offering financial incentives or subsidies to ophthalmologists who establish practices in underserved rural areas could be an effective strategy to improve access to specialized eye care and reduce health disparities in these regions.

### Educational outreach

Educational outreach initiatives hold promise for improving the delivery of care to DR patients in rural areas. These efforts target various facets of the healthcare system, each with a unique approach. One strategy involves training community members as health educators. These individuals, familiar with the cultural nuances and challenges within their communities, can provide personalized education and coaching, effectively bridging the gap between patients and clinical care [55]. Another approach focuses on partnering with rural primary care providers, ensuring that patients living with diabetes consistently receive guidance from local healthcare professionals on the importance of regular eye exams. Since rural patients often first seek care from primary care providers, these providers play a crucial role in improving eye care management [56,57]. Additionally, awareness campaigns within rural communities can boost public knowledge about DR and encourage routine screenings [58]. Such campaigns delivered through media, community-led events, and other means can promote early detection and ultimately lead to better outcomes for underserved patients.

### Enhanced training programmes

Enhancing the training of general ophthalmologists in administering intravitreal injections could significantly improve access to therapeutic options for DR in rural areas. This effort could involve several key components. First, ophthalmology residency programmes could provide more extensive training in intraocular injections, ensuring that all newly graduating ophthalmologists are proficient in this essential skill. Second, training residents in remote locations could prepare them to work effectively in settings with limited resources. Lastly, offering continuing education courses on intravitreal injections for practicing ophthalmologists could strengthen their expertise, particularly benefiting rural providers who wish to expand their service offerings. By implementing such interventions, the healthcare system can enhance access to advanced treatments in rural communities, ultimately improving patient care in these underserved areas.

### Increased rural medical school admissions

Research has shown that individuals raised in rural areas are more likely to return and practice in those communities after completing their medical education [59]. To enhance rural enrollment in medical programs, several strategies could be employed. For instance, targeting high schools and colleges in rural regions for recruitment into medical school could increase the number of future physicians with a commitment to rural healthcare.

Moreover, medical schools could develop specialized tracks focused on rural healthcare, addressing the diverse medical needs of frontier communities, including fields like ophthalmology. This could take the form of a rural health training pathway, offering students practical experience and exposure to delivering care in rural settings [60].

Mentorship programs between rural medical students and practicing rural healthcare providers throughout the different stages of medical education could also be invaluable. Such relationships may help students navigate the unique challenges of rural practice while fostering long-term dedication to underserved populations [61].

By implementing these strategies, medical schools can contribute to strengthening the workforce in underserved areas, particularly by training ophthalmologists and other specialists to meet the healthcare needs of rural communities.

### Ophthalmology residency and fellowship initiatives

Ophthalmology residency programs and retina fellowships must take a more proactive role in training and preparing candidates for careers in rural areas. This can be achieved through three key approaches. First, programs could develop specific tracks within medical school, residency, and fellowship that focus on the unique practice settings common in rural America, tailoring training to meet the needs of these communities. Second, requiring rural rotations as part of training could increase awareness among residents and fellows about the opportunities and challenges of practicing in more geographically isolated areas. These rotations would not only provide hands-on experience but also foster interest in rural healthcare research, further developing expertise in underserved regions.^60^ Through these measures, ophthalmology training programs could help alleviate regional shortages in the eye care workforce, ensuring that underserved rural populations receive high-quality vision care. This approach could result in more ophthalmologists and retina specialists eager to relocate to rural areas, directly benefiting these communities.

## Conclusion

DR remains a key health concern in rural communities who face multiple barriers in access to care including physical distance, socioeconomic factors, and provider shortages. A variety of potential solutions are available to confront these issues, such as telehealth, AI screening, and targeted education and training programmes related to workforce issues. In order to enact these solutions, however, a comprehensive multistakeholder response from policymakers, healthcare systems, educators and technology developers will be required to best serve those in rural communities.

## Data Availability

This manuscript was drafted utilizing previously published articles found in the public domain and did not generate any novel data. Therefore, data sharing is not applicable for this manuscript.
